# Gracilis muscle transposition in complex anorectal fistulas of diverse types and etiologies: long-term results of 60 cases

**DOI:** 10.1007/s00384-022-04293-6

**Published:** 2023-01-18

**Authors:** Mila Isabelle Schoene, Sabine Schatz, Marion Brunner, Alois Fuerst

**Affiliations:** 1 Department of Surgery, Caritas Clinic St. Josef, Landshuter Str. 65, 93053 Regensburg, Germany; 2 University of Regensburg, Regensburg, Germany

**Keywords:** Anal fistula, Recurrent fistulas, Gracilis muscle, Muscle transposition

## Abstract

**Purpose:**

Complex fistulas often require several attempts at repair and continue to be a challenging task for the surgeon, but above all, a major burden for the affected patient. This study is aimed at evaluating the potential of gracilis muscle transposition (GMT) as a therapeutic option for complex fistulas of diverse etiologies.

**Methods:**

A retrospective study was conducted over a period of 16 years with a total of 60 patients (mean age 50 years). All were treated for complex fistula with GMT at St. Josef’s Hospital in Regensburg, Germany. Follow-up data were collected and analyzed using a prospective database and telephone interview. Success was defined as the absence of fistula.

**Results:**

A total of 60 patients (44 women, 16 men; mean age 50 years, range 24–82 years) were reviewed from January 2005 to June 2021. Primary fistula closure after GMT was achieved in 20 patients (33%) and 19 required further interventions for final healing. Overall healing rate was 65%. Fistula type was heterogeneous, with a dominant subgroup of 35 rectovaginal fistulas. Etiologies of the fistulas were irradiation, abscesses, obstetric injury, and iatrogenic/unknown, and 98% of patients had had previous unsuccessful repair attempts (mean 3.6, range 1–15). In 60% of patients with a stoma (all patients had a stoma, 60/60), stoma closure could be performed after successful fistula closure. Mean follow-up after surgery was 35.9 months (range 1–187 months). No severe intraoperative complications occurred. Postoperative complications were observed in 25%: wound healing disorders (*n* = 6), gracilis necroses (*n* = 3), incisional hernia (*n* = 2), scar tissue pain (*n* = 2), suture granuloma (*n* = 1), and osteomyelitis (*n* = 1). In 3 patients, a second gracilis transposition was performed due to fistula recurrence (*n* = 2) or fecal incontinence (*n* = 1).

**Conclusion:**

Based on the authors’ experience, GMT is an effective therapeutic option for the treatment of complex fistulas when other therapeutic attempts have failed and should therefore be considered earlier in the treatment process. It should be seen as the main but not the only step, as additional procedures may be required for complete closure in some cases.

## Introduction

Complex fistulas continue to be a major problem in proctology. There is no precise definition for the term “complex” in this context, and it may therefore vary among authors [1, 2]. It usually refers to fistulas that are difficult to treat and prone to recurrence. This type of fistula is often caused by tissue defects due to irradiation, surgery, irritable bowel disease (IBD), or obstetric injuries. Many therapeutic options have been described [3–9], yet standardization is still lacking and no consensus regarding the best approach has been reached to date. This, in turn, means that patients often present with a history of multiple failed attempts at repair before gracilis muscle transposition (GMT)—which is often considered a last resort—is performed.

The diagnosis of a complex fistula places enormous stress and limitations on almost every aspect of the patient’s life and leads to depression, anxiety, and avoidance of medical examinations [10]. The only solution is therefore complete and definitive closure of the fistula. The gracilis muscle has been used extensively in reconstructive surgery and has achieved good results in combating fistulas due to the well-perfused tissue it provides [2, 4, 10–26]. Overall healing rates range between 71 and 100% in the literature. Because of the complexity of closing this type of fistula, it may be beneficial to move towards a more aggressive approach in the future, rather than following the common approach of steadily escalating therapy in order to improve surgical conditions and chances of success. This study is aimed at contributing to the analysis of GMT as an effective therapeutic option in patients with complex fistulas.

## Methods and patients

A total of 60 patients (44 women, 16 men; mean age 50 years) undergoing GMT between January 2005 and June 2021 at the authors’ institution in Regensburg, Germany, for treatment of complex rectourethral, rectovesical, anovaginal, pouch-vaginal, urethrocutaneous, vesicovaginal, urethrovaginal, rectovaginal, and anal fistulas were analyzed. Patients were divided into four groups reflecting the heterogeneous etiologies of the fistulas: (1) irradiation, (2) consequence of current or previous abscesses, (3) obstetric injuries, and (4) iatrogenic/unknown. Fistulas caused by IBD were excluded. The results of GMT of IBD patients were published previously. GMTs for the treatment of anal incontinence without any findings of a fistula were also excluded from the study.

Results on GMT performed due to IBD fistulas at the authors’ institute have already been published by Fürst et al. and Korsun et al. [15, 25]. Data including demographics, etiologies, age at the time of surgery, type of fistula, follow-up (months), previous repair attempts, stoma closure, and postoperative complications were recorded. Outcomes and follow-up examinations were evaluated and documented in a prospective database. Some patients were additionally interviewed by telephone to obtain follow-up data if necessary. Both the primary healing rate after GMT and the overall healing rate, i.e., when recurrence occurred and additional procedures were required for complete healing, were evaluated.

### Surgical technique

**Fig.1:**
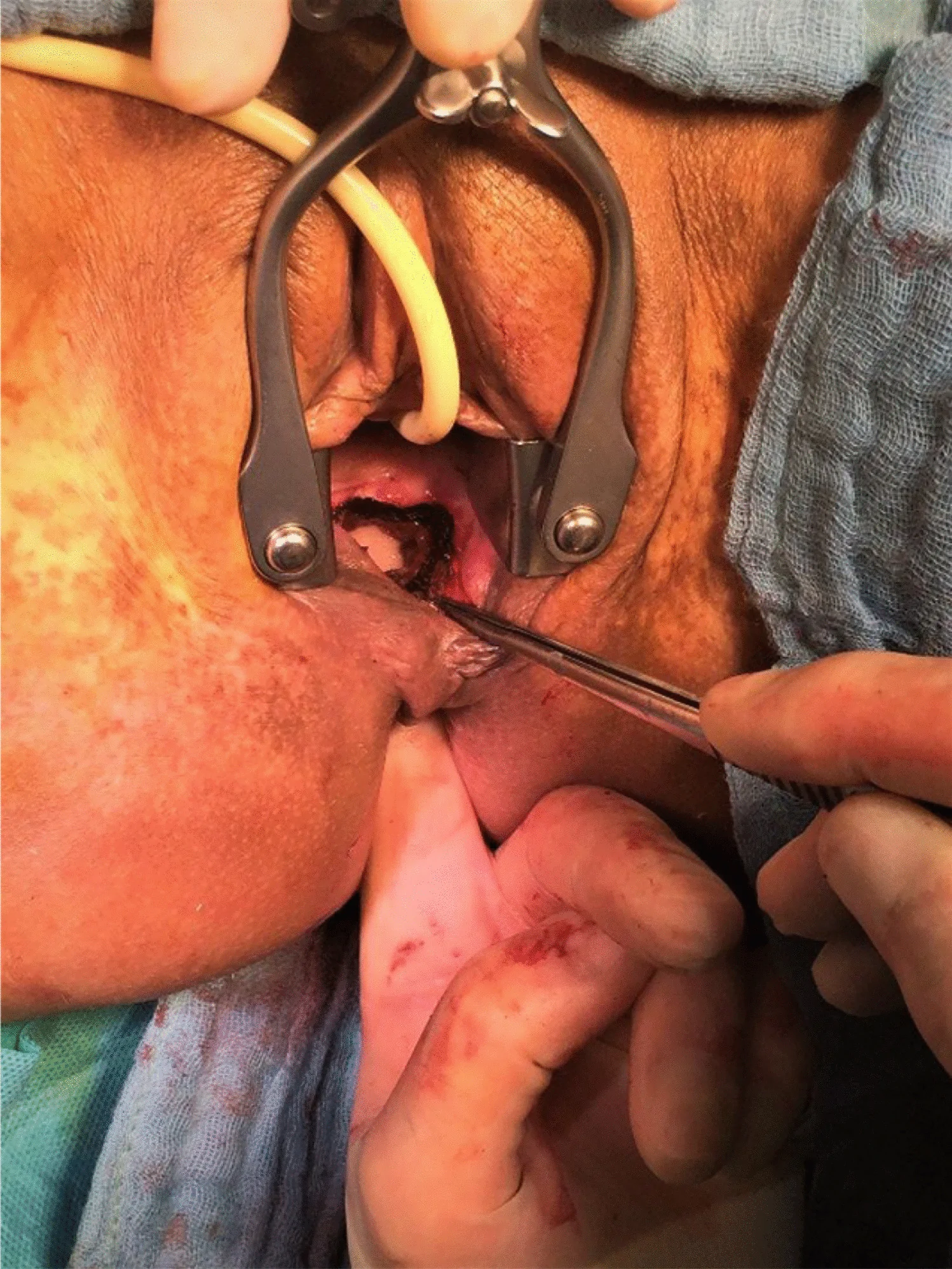
Complex rectovaginal fistula after irradiation of an anal cancer

**Fig. 2:**
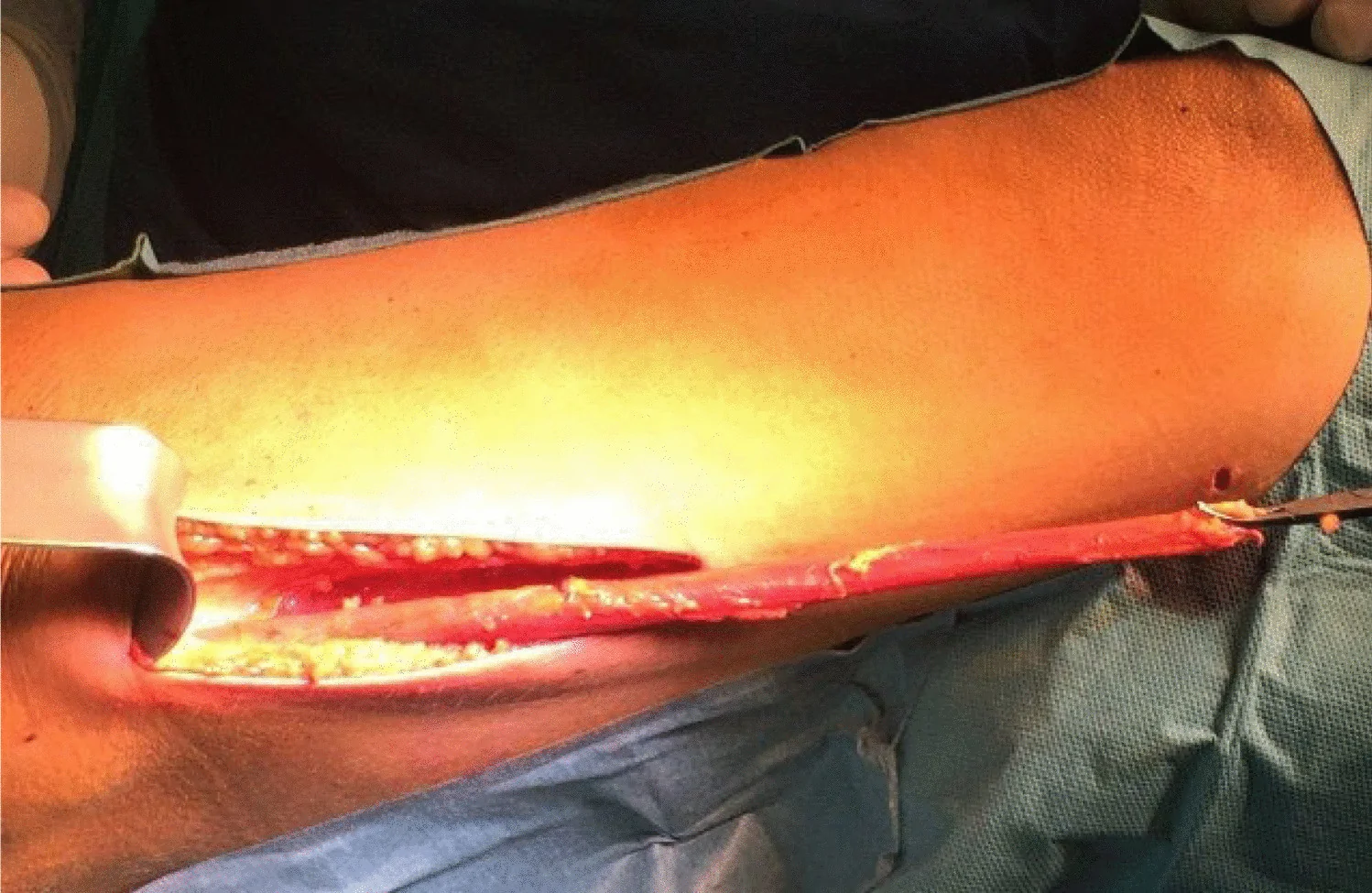
Incisions on thigh and mobilization of the gracilis

**Fig. 3:**
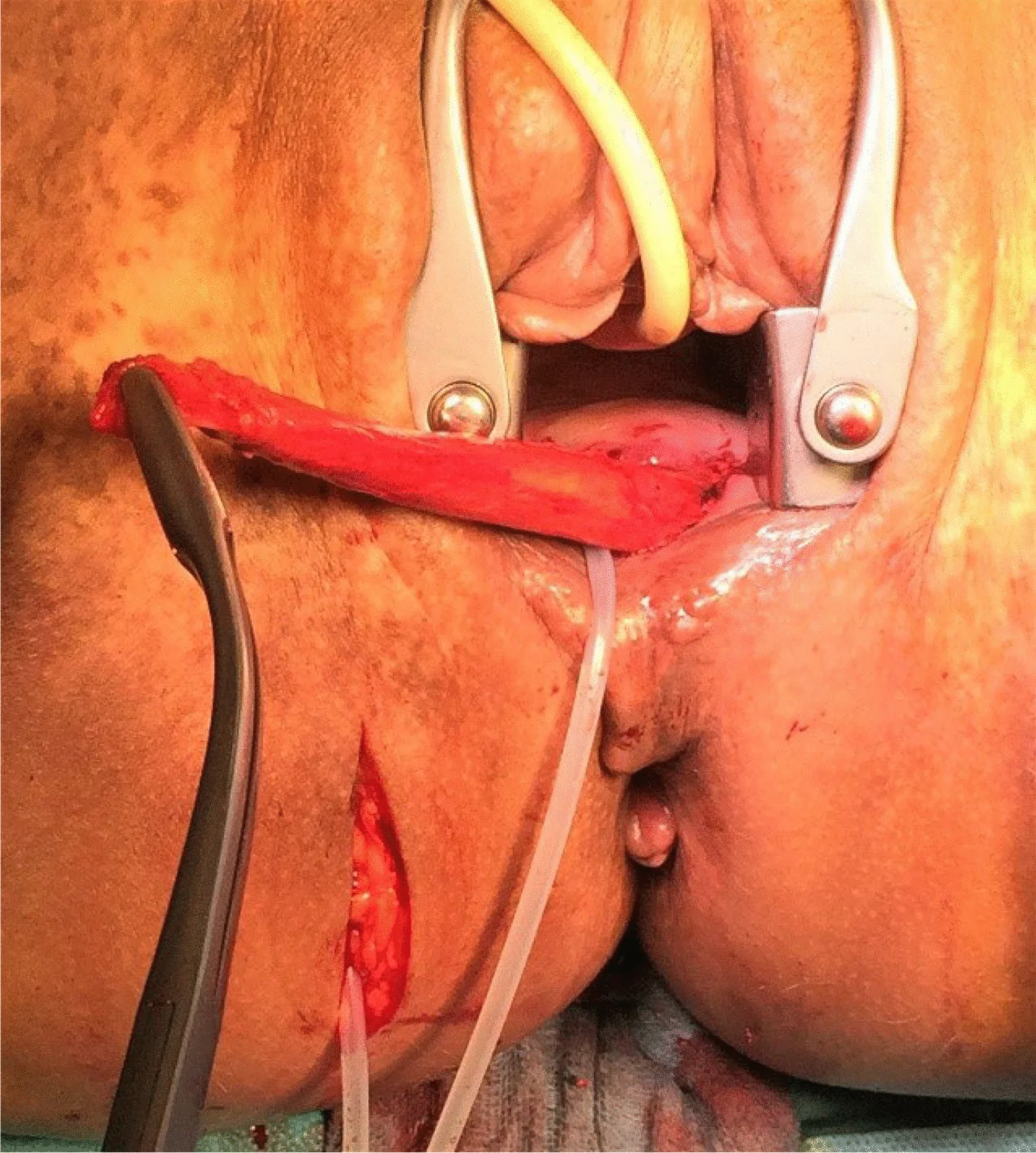
First tunneling of the gracilis muscle and positioning at the level of removed fistula

**Fig. 4:**
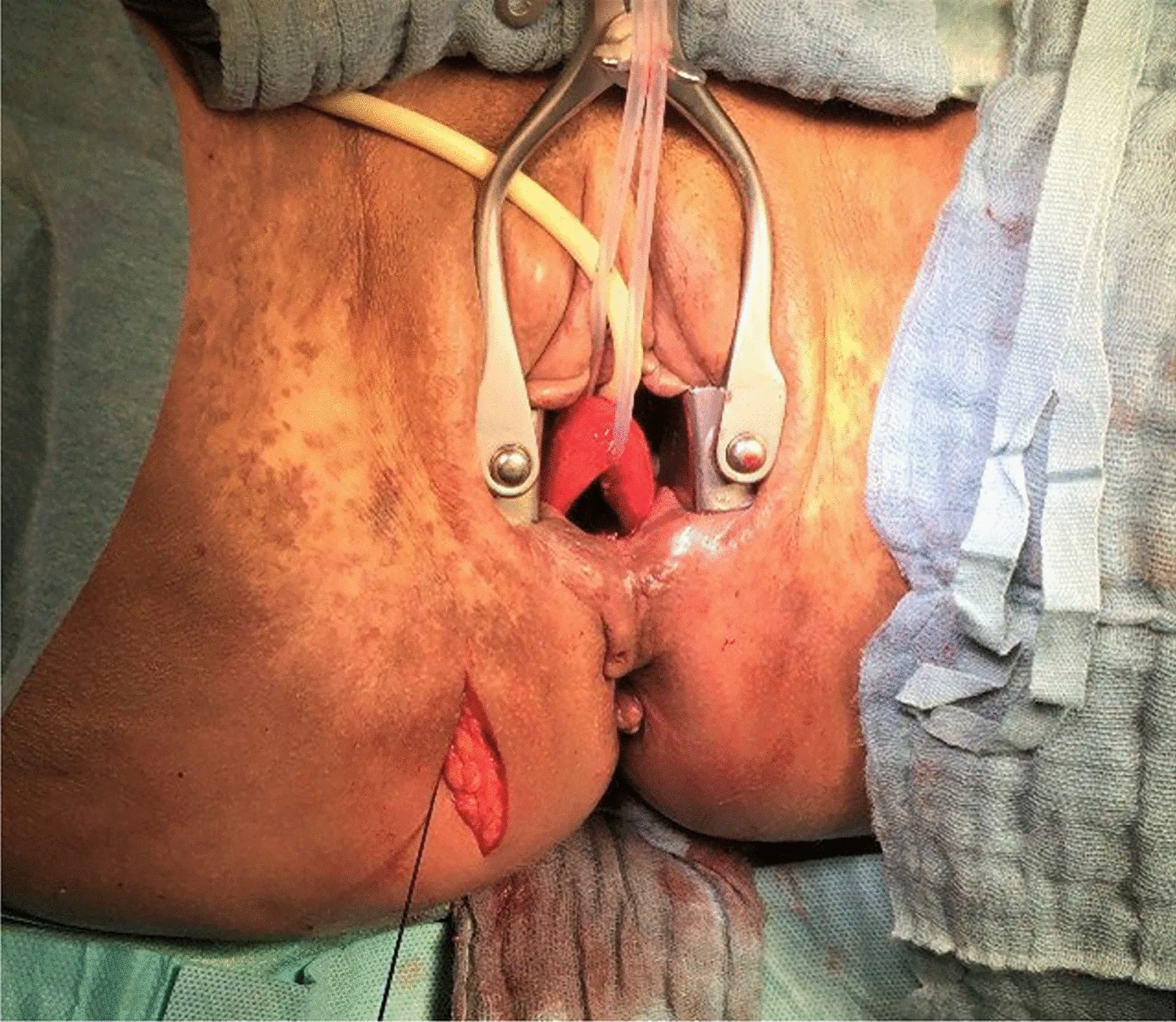
Second tunneling of the gracilis muscle and fixation to the contralateral periosteum with lateral incision

**Fig. 5:**
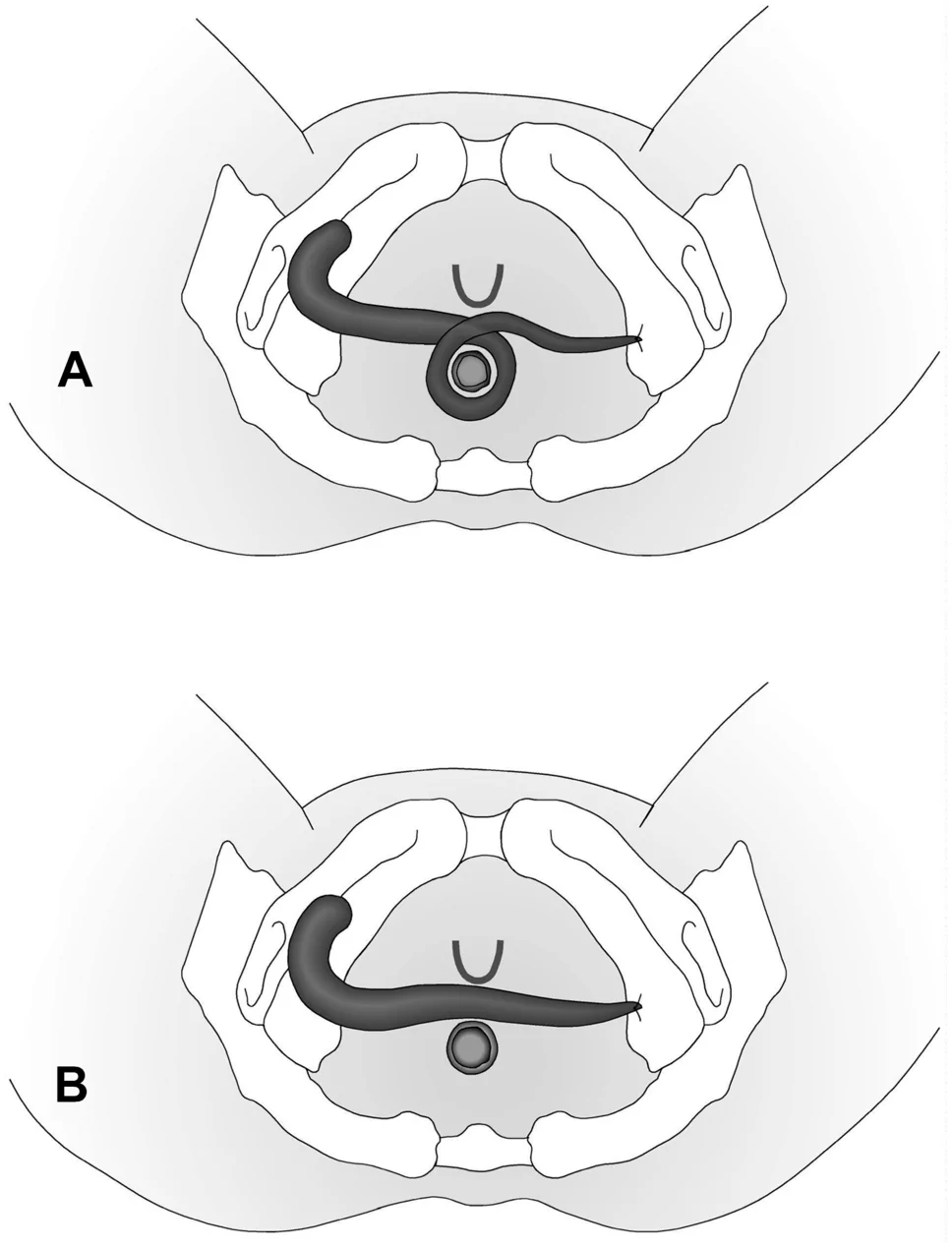
**A** Transverse GMT. **B** Circular GMT

GMT was performed at the authors’ institution according to the tunnel technique of Baeten et al. [27], as previously described in detail by Kersting et al. and Fuerst [24, 28]. In brief, a stoma was established in all patients preoperatively or at the time of GMT to create the best possible healing conditions. Antibiotics were administered and the patient was placed in a lithotomy position under general anesthesia. The fistula tract and its openings were thoroughly removed (Fig. 1). The gracilis muscle was then identified, and two incisions were made along the thigh, a larger one of about 8–10 cm in the medial compartment and a smaller one of 2 cm at the knee, near the pes anserinus (Fig. 2). The muscle was mobilized, small irrelevant blood vessels were ligated, and the muscle was rotated. Its distal end was pulled through a bluntly prepared tunnel between the proximal incision and the perineum. Great care was taken to work in a tension-free manner during this step, to avoid damage to the vessels or nerve supply to the muscle. The muscle was finally placed at the level of the removed fistula, filling the defect with well-vascularized tissue (Fig. 3). The gracilis was then attached to the periosteum of the contralateral pubic bone or the ischium with Prolene sutures (Ethicon Inc.) by pulling it again through a second prepared subcutaneous tunnel and making a small lateral incision (Fig. 4). If the patient also suffered from anal incontinence, the muscle was looped around the anal canal and attached to the ipsilateral periosteum (Fig. 5). In this way, a neosphincter was created that provided the anal canal with healthy muscle tissue, narrowing and strengthening it. In addition, there was the option of secondary dynamization by implanting stimulating electrodes to further control sphincter function [27].

In the authors’ experience, three steps are necessary to achieve maximum success: firstly, the tunneling must be wide enough to enable pressure-free positioning of the muscle; secondly, positioning of the muscle on or slightly above the removed fistula is essential for healing of the area; and thirdly, incisions in the perineum are to be avoided, as wound healing in this region can be impaired.

### Outcome measures

Primary outcome was the primary healing rate, defined as definitive fistula closure after GMT without recurrence within the first 3 postoperative months. Common fistula symptoms such as vaginal flatulence, fecaluria, abnormal discharge, or any signs of infection were observed. In addition, all patients received further clinical diagnostics, including air insufflation, urethrocystoscopy, proctoscopy, gynecological examination, and, if necessary, probing of the fistula tract. In cases where the presence or absence of the fistula could not be clearly proven, anorectal endosonography, water-soluble contrast enema, or magnetic resonance imaging of the pelvis could additionally be performed. Secondary outcome was the overall healing rate, i.e., including the patients who had required further procedures for definitive fistula closure by the time of data collection.

### Stoma closure

If healing of the fistula could be proven by clinical examination, stoma closure was performed.

## Results

A total of 63 GMTs were performed in 60 patients (44 women, 16 men) between January 2005 and March 2021. Mean age at the time of surgery was 50 years (range 24–82 years). The underlying events promoting a fistula were (1) irradiation (*n* = 17), (2) abscess (*n* = 14), (3) obstetric injury (*n* = 8), and (4) iatrogenic/unknown (*n* = 21; Table 1). Mean follow-up after surgery was 35.9 months (range 1–187 months). The following types of fistulas were documented: rectourethral, rectovesical, anovaginal, pouch-vaginal, urethrocutaneous, vesicovaginal, urethrovaginal, rectovaginal, and anal (Table 2). Of the total patients, 59 had undergone previous unsuccessful repair attempts (mean 3.6, range 1–15). Only one irradiated patient with a rectovaginal fistula had no previous closure attempts and was successfully treated with GMT. Complete closure was achieved primarily by GMT in 20 patients (33%). In 19 patients, further treatment was required for definitive closure, ultimately corresponding to an overall healing rate of 65% (Table 2). Whereas 23 patients (38%) developed early recurrence within the first 3 postoperative months (Table 3), late recurrence occurred in 17 patients (28%). In 3 patients, repeat GMT of the opposite side was required due to recurrence (in 1 patient, the first interposition was performed at another institution). In 2 patients, the second gracilis interposition resulted in healing, while in the other case, the fistula was still present at the time of data collection. A fourth patient received a second dynamic graciloplasty not due to recurrence, but because of fecal incontinence caused by massive tissue defects due to multiple previous attempts at repair. The highest overall healing rate (88%) was documented in group 3 (obstetric injuries; Table 3). This is also the group with the lowest number of previous repair attempts (mean 1.9, range 1–4). The second highest success rate was achieved in group 4 (iatrogenic/unknown). In this group, the number of previous failed repairs was significantly higher compared to groups 3 and 1 (mean 4.2, range 2–15). The success rate here increased from 33% after GMT alone to 76% after further interventions. Group 4 is also the largest group in our series, with a total of 21 patients who developed a fistula after pelvic interventions for various indications. In 3 cases also belonging to this group, the exact etiology remained unknown. Irradiated patients had a primary healing rate of 35% and an overall healing rate of 47%, the lowest success rates in our study. An average of 2.1 (range 1–10) previous repair attempts were observed here. The highest mean number of previous repair attempts was recorded in group 2 (abscess; mean 4.9, range 2–15); 3 patients (21%) were cured here primarily by GMT and 5 others after additional procedures, to give an overall healing rate of 57% in this group. In all 60 patients, a stoma was established preoperatively or at the time of surgery, which could be reversed in 36 cases. A total of 15 complications (25%) were documented, including 6 cases of wound healing disorders, 3 necroses of the gracilis muscle, 2 incisional hernia, 2 cases of scar tissue pain, 1 suture granuloma, and 1 case of osteomyelitis (Table 4).

**Table 1:** Etiology of recurrent fistulas in 60 patients

Etiology	*n* (%)
Irradiation	17 (28.3)
Abscess	14 (23.3)
Obstetric injury	8 (13.3)
Iatrogenic/unknown	21 (35)

**Table 2:** Collected data *n* (%)

Data	(1) Irradiation	(2) Abscess	(3) Obstetric injury	(4) Iatrogenic/unknown	Total
Number of patients	17 (28.3)	14 (23.3)	8 (13.3)	21 (35)	**60 (100)**
Mean age (years)	58.9	45.4	42.3	48.1	50
Mean follow-up (months)	43.4	31.5	24.3	36.4	36
Patients with previous attempts at repair	16 (10.2)	14 (23.7)	8 (14.6)	21 (35.6)	59 (98.3)
Primary healing	6 (30)	3 (15)	4 (20)	7 (35)	20 (33.3)
Overall healing	8 (18.4)	8 (26.3)	7 (18.4)	16 (36.8)	39 (65)
Stoma	17 (28.3)	14 (23.3)	8 (13.3)	21 (35)	60 (100)
Stoma closure	7 (19.4)	7 (19.4)	6 (16.7)	16 (44.4)	36 (60)
Fistulas					**61**
Rectovaginal	8	7	6	14	35
Pouch-vaginal	-	-	-	1	1
Anovaginal	-	-	1	2	3
Rectourethral	2	-	-	1	3
Rectovesical	4	-	-	-	4
Urethrovaginal	-	-	-	1	1
Urethrocutaneous	1	-	-	-	1
Vesicovaginal	1	-	-	-	1
Anal	2	7	1	2	12

(%) in a row refers to total number in the last row. (%) in the last column refers to the total number of patients (*n* = 60)

**Table 3:** Outcome of 60 patients *n*/*n* (%)

Outcome	(1) Irradiation	(2) Abscess	(3) Obstetric Injury	(4) Iatrogenic/unknown
Median attempts at repair	2.1	4.9	1.9	4.2
Recurrence (3 months)	7/17 (41.2)	7/14 (50)	1/8 (12.5)	8/21 (38.1)
Late recurrence	4/17 (23.5)	4/14 (28.6)	3/8 (37.5)	6/21 (28.6)
Primary healing	6/17 (35.3)	3/14 (21.4)	4/8 (50)	7/21 (33.3)
Overall healing	8/17 (47.1)	8/14 (57.1)	7/8 (87.5)	16/21 (76.2)

**Table 4:** Postoperative complications in 60 patients

Complications	*n*
Necrosis of the gracilis muscle	3
Wound healing disorder	6
Incisional hernia	2
Scar tissue pain	2
Suture granuloma	1
Osteomyelitis	1
Total	15

## Discussion

Complex fistulas have a higher tendency to recur and remain a challenge for both the surgeon and the patient. Several treatment options have been described [3–9], but no consensus or standard regarding the best possible treatment has been reached to date. As a result, patients often have a history of multiple failed attempts before GMT is performed [22]. Although gracilis muscle interposition was first described in 1928 [29], it is not performed in every institution and is therefore not considered to be an early therapeutic option in many cases.

In the current study, 63 GMTs were performed in 60 patients. Patients were divided into four groups according to fistula etiology. The results show a primary healing rate of 33% and an overall healing rate of 65% when further intervention was indicated for final closure of the fistula. Similar results were obtained in previous studies [2, 4, 10–26]. Success rates in previous studies varied from 42 to 100% for primary healing by GMT alone and from 71 to 100% for overall healing (Table 5). The lower healing rates in the present series compared to previous studies might be explained by the high heterogeneity of fistula causes, particularly in group 4 (iatrogenic/unknown), the different fistula types, and the relatively high number of cancer patients, who are generally associated with lower healing rates [30–33].

**Table 5:** Overview of previous studies (2000–2022)

Author	Year	Number of patients	Etiology	Previous repair attempts (mean)	Primary healing (%)	Overall healing (%)
Maeda et al. [11]	2001	14	CD	n.m. in detail	64	n.m
Zmora et al. [12]	2003	11	Iatrogenic	n.m. in detail	83	100
Zmora et al. [13]	2006	9	Heterogeneous	n.m. in detail	88	n.m
Wexner et al. [14]	2008	53	Heterogeneous	1.5 and 2	75	97
Fürst et al. [15]	2008	12	CD	n.m. in detail	92	n.m
Ulrich et al. [16]	2009	35	Heterogeneous	n.m. in detail	94	n.m
Lefèvre et al. [17]	2009	8	Heterogeneous	3	75	88
Pinto et al. [18]	2010	25	Heterogeneous	n.m. in detail	79	n.m
Nassar [19]	2011	11	Pelvic surgery	n.m. in detail	100	-
Chen et al. [20]	2013	11	Heterogeneous	1	74	95
Troja et al. [21]	2013	10	Heterogeneous	n.m. in detail	60	n.m
Corte et al. [4]	2015	32	Heterogeneous	n.m. in detail	50	n.m
Park et al. [22]	2017	11	Heterogeneous	1	73	n.m
Rottoli et al. [23]	2018	13	Heterogeneous	2	68	n.m
Rottoli et al. [23]	2018	8	IBD	1	75	n.m
Kersting et al. [24]	2019	19	Heterogeneous	4	53	76
Korsun et al. [25]	2019	32	IBD	2	47	71
Grott et al. [2]	2021	46	Heterogeneous	2	53	74
Yellinek et al. [26]	2021	119	Heterogeneous	n.m. in detail	42	92
Maljaars et al. [10]	2022	18	Obstetric injury	n.m. in detail	83	n.m
Recent study	2022	60	Heterogeneous	3.6	33	65

*CD* Crohn’s disease, *IBD* inflammatory bowel disease, *n.m.* not mentioned

The number of comparable studies investigating the efficacy of GMT in controlling fistulas is still small (Table 5). In 2003, Zmora et al. [12] reported a healing rate of 83% in 11 men with rectourethral fistulas. In 2 patients, further treatment was required for final fistula closure; in 5 patients, fistula repair had been attempted previously. In another series 3 years later, the authors described fistula closure in 88% of 9 patients [13]. Ulrich et al. documented successful fistula closure in 32 cases (94%) [16]. In 2009, Wexner et al. [14] reported a final healing rate of 97% in a larger study with 53 cases; the initial healing rate without further intervention was 78%. What almost all these studies have in common is the fact that a significant increase in the success rate was observed when further procedures were performed after GMT. This was shown particularly clearly in a recent study by Yellinek et al. [26], which is also the largest series to date with a total of 119 patients. The percentage of successfully closed fistulas in the latter study increased from 42 to 92% after additional interventions.

The diagnosis of a complex fistula represents an enormous burden for patients. Restrictions, social stigmatization, and isolation are often part of their everyday life [10, 24, 34]. The fact that several failed attempts are common exacerbates patients’ suffering. In 2021, Grott et al. [2] analyzed outcomes and quality of life after GMT in 47 cases. The overall healing rate increased from 53 to 74% with further interventions. They asked patients if they would recommend GMT to other patients. All patients in whom closure had been achieved answered in the affirmative. Of those who had a recurrence, none would recommend the method. This underscores the fact that the only goal should be definitive closure of the fistula. In a series by Kersting et al. [24], the focus was also on the change in quality of life, with 11 patients (58%) reporting an improvement after GMT. Fistula closure was achieved in 76% (*n* = 14).

The gracilis muscle has two main advantages for the healing process. First, the muscle fills defects often created by trauma, surgery, or inflammation in complex fistulas, and second, its blood supply ensures healthy, well-perfused tissue in areas where this is no longer present. The current series includes a heterogeneous group of patients and fistulas (Table 4). The best results were obtained in group 3 (obstetric injuries), with 7 of 8 fistulas closed. Grott et al. [2] reported a 100% healing rate for the same number of obstetric fistulas. These results are consistent with the findings of Maljaars et al. [10], who reported on 15 (83%) obstetric fistulas successfully closed by GMT between 2006 and 2009 at the Fistula Closure Centre in Malawi. The Singapore flap was successful in 14 cases (54%) and a combination of both flaps in 50% (*n* = 8). In the present series, obstetric injuries are also the group with the lowest number of repair attempts before GMT (mean 1.9).

The number of previous attempts plays an important role in healing and success [18, 22]. Lowry et al. found that the success rate dropped by more than 30% after two failed repair attempts [35]. In the current series, group 2 (abscesses) had undergone a mean of 4.9 prior attempts to close the fistula and had the highest recurrence rate after GMT of 79%. The initial closure rate after GMT was increased by 35% with further interventions. Group 4 (iatrogenic/unknown) also had a significantly higher number of prior failed attempts (mean 4.2); however, the second-best healing rate in our series was achieved in this group, with 76%. This demonstrates the effectiveness of transferring healthy, well-vascularized muscle flaps to damaged tissue. In 2017, Park et al. reported a lower recurrence rate in patients with a higher number of prior surgeries who underwent graciloplasty compared to a control group with a lower number of previous surgeries but who underwent other surgical procedures for fistula closure [22].

Treatment of radiogenic fistulas remains difficult. In the present study, this is the group with the lowest overall healing rate (47%). Previous studies have confirmed that patients who have undergone radiotherapy tend to require more repair attempts due to excessive tissue damage [31–33]. Kocot and Riedmiller stated that improvement can only be achieved by adequate blood perfusion [30].

The current study has several limitations, firstly due to its retrospective nature and the heterogeneous etiologies and types of fistulas. Secondly, the number of patients in some groups is small. In addition, it is not possible to accurately measure the contribution of GMT to the overall healing process if further procedures were required to close the fistula [25]. More standardization and further research are needed to investigate this aspect. However, it appears that the gracilis muscle, although not the sole contributor to success in some cases, paves the way for the healing process.

The approach to this type of fistula must therefore be understood as multi-stage. Rather than attempting to close complex fistulas through various types of surgery, the very first thing that needs to be done is to create a good starting point for successful surgery and healing. This represents healthy, well-perfused tissue, which the gracilis can provide. Subsequently, further conventional operations can follow to close the fistula finally.

In our series, the healing rate increased by 12–43% after further procedures. It is therefore important to clearly communicate to patients preoperatively that while GMT forms the basis for fistula closure, follow-up procedures may be part of the process, particularly in patients who have undergone multiple previous operations, and that this should not be seen as another failed attempt. This prevents false expectations and improves patient compliance in this very sensitive matter.

In the present study, the complication rate was 25%. Considering the serious impact that this condition can have on people’s lives, we believe that complex fistulas should be addressed more aggressively at an early stage. This creates better surgical conditions, makes the journey less traumatic for affected patients, and can potentially reduce the tendency for recurrence. In 2022, Park et al. published a study in which surgical closure of rectourethral fistulas had been performed and observed that significantly fewer recurrences occurred in patients (*n* = 24) treated with gracilis interposition compared to a control group [36]. The current authors’ approach is in line with the statement of Corte et al. [4], who analyzed different treatment management strategies in 79 patients with rectovaginal fistulas. They concluded that in recurrent fistulas, the step-up approach should be changed to more invasive, major interventions. Other authors agreed [13, 21, 22].

## Conclusion

Gracilis muscle transposition is, in the authors’ experience, an effective treatment for complex fistulas, especially after obstetric trauma. This treatment option should be considered much earlier and must be understood with the healthy tissue provided by the gracilis muscle. Our study shows that graciloplasty is the basis of treatment and healing process, but not necessarily the step alone to achieve final fistula closure.
